# Role of Immunotherapy in Stage IV Large Cell Neuroendocrine Carcinoma of the Lung

**DOI:** 10.31557/APJCP.2021.22.2.365

**Published:** 2021-02

**Authors:** Takefumi Komiya, Neema Ravindra, Emily Powell

**Affiliations:** 1 *Medical Oncology, Parkview Cancer Institute, 11050 Parkview Circle, Fort Wayne, IN 46845, USA. *; 2 *Parkview Research Center, Mirro Center for Research and Innovation, 3948- A New Vision Drive, Fort Wayne, IN 46845, USA. *

**Keywords:** Immunotherapy, large cell neuroendocrine carcinoma, lung cancer

## Abstract

**Background:**

Despite approvals of immune checkpoint inhibitors in both small cell and non-small cell lung cancers, the role of immunotherapy in large cell neuroendocrine carcinoma (LCNEC) in lung is undefined.

**Methods:**

Using the National Cancer Database (NCDB), Stage IV lung LCNEC cases diagnosed from 2014 to 2016 were analyzed. Information regarding cancer treatment was limited to first course of therapy, including surgery for primary lesion, radiation, chemotherapy, and immunotherapy. Survival analysis was performed using Kaplan-Meier curves and Log-rank tests. Cox proportional hazard model was used for multivariate analysis.

**Results:**

Among 661 eligible cases, 37 patients were treated with immunotherapy. No significant association between use of immunotherapy and clinical demographics was observed except for use of chemotherapy (p=0.0008). Chemotherapy was administered in 34 (92%) and 406 (65%) in immunotherapy and non-immunotherapy groups, respectively. Use of immunotherapy was associated with improved overall survival (Log-rank p=0.0018). Landmark analysis in the immunotherapy group showed 12 and 18-month survivals of 34.0% and 29.1%, respectively, whereas those in the non-immunotherapy group were 24.1% and 15.0%, respectively. Multivariate analysis demonstrated that female sex (HR=0.79, p=0.0063), liver metastases (HR=0.75, p=.0392), surgery (HR= 0.50, p<0.0001) use of chemotherapy (HR= 0.44, p<0.0001), and use of immunotherapy (HR=0.64, p=0.0164) had statistical significance. Propensity score matching in overall survival analysis showed a nonsignificant trend (p=0.0733) in favor of immunotherapy treatment.

**Conclusion:**

This retrospective study using NCDB suggests that use of immunotherapy may improve survival of LCNEC patients.

## Introduction

Large cell neuroendocrine carcinoma (LCNEC) is a relatively rare histologic subtype, accounting for approximately 3% of lung cancer cases (Fasano et al., 2015, Rekhtman, 2010). It has been described in recent decades since Travis et al. originally described it in the early 1990s (Travis, et al., 1991). Despite its neuroendocrine features, it was initially classified as a variant of large cell carcinoma by the 2004 World Health Organization (WHO). The current 2015 version of WHO lists it in a group of neuroendocrine neoplasms along with typical carcinoid, atypical carcinoid, and small cell carcinoma (Travis et al., 2015).

Due to its sparsity of cases and difficulty in diagnosis with small biopsy samples, standard systemic therapy for advanced stage has not been well established. To our knowledge, no prospective, randomized study comparing multiple systemic regiments has been reported in the literature. Limited literature with retrospective studies, case reviews, and single arm prospective trials suggest that regimens used for small cell lung cancers (i.e., platinum plus etoposide) are superior to those used for non-small cell lung cancer (i.e., platinum plus taxane) and result in improved patient outcomes for stage IV cancers, as well as for early stages when the chemotherapy is given as adjuvant therapy (Sun et al., 2012, Zhang et al., 2020, Le Treut et al., 2013, Niho et al., 2013) although contradictory reports exist (Derks et al., 2017).

Recent progress in the development of immune checkpoint inhibitors has dramatically changed survival outcomes and disease management for lung cancer. Several agents have been approved as monotherapy or are used in combination with chemotherapy agents for a number of human cancer types. Pembrolizumab, nivolumab, atezolizumab, and durvalumab are approved by the FDA for the treatment of advanced non-small cell lung cancer (NSCLC). Treatment with atezolizumab or durvalumab, a monoclonal antibody directed against programmed cell death ligand 1 (PDL1), resulted in improved overall survival of patients with extensive stage small cell lung cancer (ES-SCLC) when combined with first-line chemotherapy (Horn et al., 2018, Paz-Ares et al., 2019). Although these agents are currently investigated in early stage settings of SCLC and NSCLC, rare cancer subtypes such as LCNEC may not be investigated soon due to paucity of the disease. It seems unlikely that controlled randomized studies will be conducted specifically for LCNEC for the next few decades. 

Because of limitations in retrospective case series and lack of potential for prospective clinical trials for rare disease such as LCNEC, cancer researchers commonly use large databases to analyze rare cancer types. Although there are some limitations, this approach allows assessment of prognosis and impact of therapeutic interventions across a larger patient population. Using the National Cancer database (NCDB), we investigated whether the use of immunotherapeutic agents influences overall survival in patients with stage IV LCNEC.

## Materials and Methods

*NCDB*

The National Cancer Data Base (NCDB) is a joint project of the Commission on Cancer (CoC) of the American College of Surgeons and the American Cancer Society. The CoC’s NCDB and the hospitals participating in the CoC NCDB are the source of the de-identified data used herein; they have not verified and are not responsible for the statistical validity of the data analysis or the conclusions derived by the authors. The data is considered as hospital-based rather than population-based.

**Figure 1:**
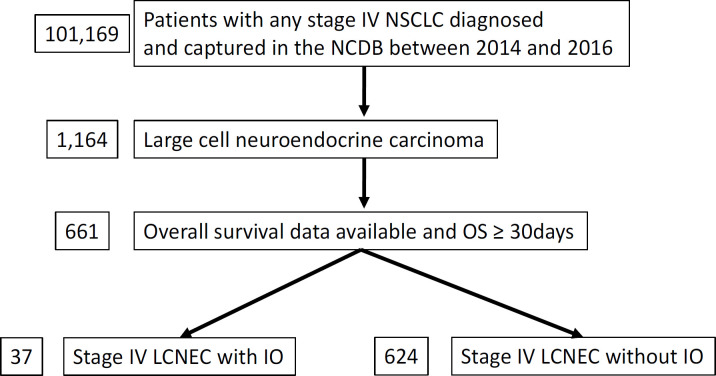
Study Flow Diagram of Case Eligibility. NSCLC, non-small cell lung cancer; NCDB, National Cancer Database; OS, overall survival; LCNEC, large cell neuroendocrine carcinoma; IO, immunotherapy

After obtaining approval by CoC, access to information of deidentified cases with stage IV NSCLC was granted in October 2019. A total of 101,169 adult cases diagnosed between 2014 and 2016 at the CoC participating institution in the United States were screened for the current study. Eligible cases must have the diagnostic ICD-O-3 code for LCNEC (8013/3) and have survived for at least one month (Figure 1). Presence or absence of IO (immunotherapy) as the first course of therapy was available. They were assigned into IO positive vs. negative groups. Information regarding name, regimen, dose, dosing frequency of IO was not available. 

Available background characteristics included age (<70 vs. 70+), sex (male vs. female), race (white vs. others), type of institution (academic vs. non-academic), presence of insurance, Charlson-Deyo comorbidity score (0-1 vs. 2-3), presence of brain/liver metastases, use of external beam radiation, use of multiagent chemotherapy in first course of therapy. Reporting any cell with less than 10 cases were prohibited according to agreement with CoC and NCDB.

Overall survival data was available according to IO status in first course of therapy. Progression-free, time-to-progression, or other survival data were not available.

*Statistics*

Relationships between clinical characteristics and use of IO were determined by chi-square tests. Survival analysis was conducted using Kaplan-Meier and Logrank methods. A p-value of less than 0.05 on a two-tailed statistical analysis was considered significant. Univariate and multivariate Cox proportional hazard analyses were performed using JMP version 14 (SAS Institute, Cary, NC, USA). Propensity score matching analysis included all the variables listed in Table 1 and was performed according to XLSTAT software guideline (Rosenbaum, 1989). 

This is a hospital-based study that involves no identifiable information for individuals throughout the analyses. This study was reviewed by the institutional review board at Parkview Health and was designated exempt from human subject research.

## Results

*Patient characteristics*

A total of 661 patients with stage IV LCNEC diagnosed between 2014 and 2016 met eligibility for this study (Table 1). Among those, 37 and 624 patients were assigned to IO or non-IO group, respectively. 

In the IO group, the majority of cases were categorized as follows: less than age 70 (68%), male sex (62%), white, insured (100%), treated at non-academic centers (54%), Charlson-Deyo (CD) comorbidity score of 0-1, absence of brain metastasis, absence of liver metastasis (73%), absence of surgery (97%), and presence of chemotherapy. There was no significant association between the clinical characteristics and presence/absence of IO except for use of chemotherapy; more patients (92%) in the IO group received chemotherapy than those in the non-IO group. In the propensity matched analysis, all the variables were matched and balanced. No significant correlation between any variable and IO status was observed (Table 2). Due to the restriction by CoC and NCDB, cells with less than 10 cases are not provided in Table 1 or 2.

*Survival analysis*

**Figure 2:**
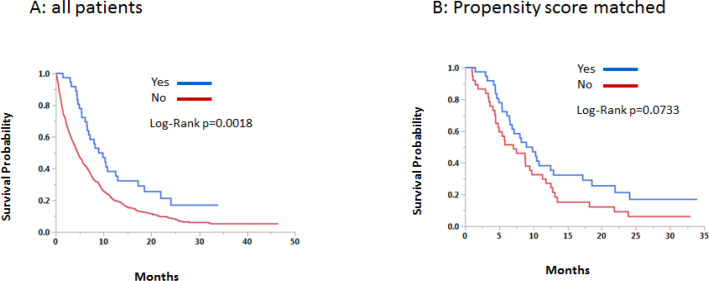
Overall Survival According to Use of Immunotherapy

Univariate and multivariate analyses were conducted for the original cohort. In the univariate analysis, significantly improved survival was seen in young age, female sex, non-white race, academic institution, CD score of 0-1, absence of liver metastasis, use of surgery, use of chemotherapy, and use of IO. Female sex, absence of liver metastasis, use of surgery, use of chemotherapy, and use of IO remained statistically significant in multivariate analyses (Table 3). Kaplan-Meier and Logrank methods demonstrated a statistically improved survival in the original cohort (p= p=0.0018) and a non-significant trend in propensity score matched cohort (p=0.0733) (Figure 2).

**Table 1:** Clinical Characteristics of Stage IV LCNEC Patients with or without Immunotherapy

	Immunotherapy		Total	P value
	Yes	No		
Total	37 (100%)	624 (100%)	661 (100%)	
Age				
<70	25 (68%)	398 (64%)	423 (64%)	0.641
70+	12 (32%)	226 (36%)	238 (36%)	
Sex				
Male	23 (62%)	337 (54%)	360 (54%)	0.333
Female	14 (38%)	287 (46%)	301 (46%)	
Race				
Whites and others	37 (100%)	624 (100%)	661 (100%)	0.943
Insurance status				
Uninsured	0 (0%)	19 (3%)	19 (3%)	0.281
Others	37 (100%)	605 (97%)	642 (97%)	
Institution				
Academic	17 (46%)	201 (32%)	218 (33%)	0.084
Others	20 (54%)	423 (68%)	443 (67%)	
Charlson-Deyo score				
0-1 and 2-3+	37 (100%)	624 (100%)	661 (100%)	0.541
Brain metastasis				
Yes and No	37 (100%)	624 (100%)	661 (100%)	0.127
Liver metastasis				
Yes	10 (27%)	170 (27%)	180 (27%)	0.977
No	27 (73%)	454 (73%)	481 (73%)	
Surgery for primary lesion				
Yes	10 (3%)	170 (6%)	180 (27%)	0.43
No	27 (97%)	454 (94%)	481 (73%)	
Radiation				
Yes	17 (46%)	324 (52%)	341 (52%)	0.48
No	20 (54%)	300 (48%)	320 (48%)	
Chemotherapy				
Yes and No	37 (100%)	624 (100%)	661 (100%)	0.0008

Note: Due to NCDB agreement, cells with less than 10 cases in Race, Charlson-Deyo score, Brain metastasis and Chemotherapy were combined with other opposing cells.

**Table 2:** Clinical Characteristics of Stage IV LCNEC Patients with or without Immunotherapy: Propensity Score Matched Cases

	Immunotherapy		Total	P value
	Yes	No		
Total	37 (100%)	37 (100%)	74 (100%)	
Age				
<70	25 (68%)	26 (70%)	51 (69%)	0.8017
70+	12 (32%)	11 (30%)	23 (11%)	
Sex				
Male	23 (62%)	25 (68%)	48 (65%)	0.6263
Female	14 (38%)	12 (32%)	26 (35%)	
Race				
Whites and Others	37 (100%)	37 (100%)	74 (100%)	0.7438
Insurance status				
Uninsured	0 (0%)	0 (0%)	0 (0%)	1
Others	37 (100%)	37 (100%)	74 (100%)	
Institution				
Academic	17 (46%)	16 (43%)	33 (45%)	0.8151
Others	20 (54%)	21 (57%)	41 (55%)	
Charlson-Deyo score				
0-1 and 2-3+	37 (100%)	37 (100%)	74 (100%)	0.1647
Brain metastasis				
Yes and No	37 (100%)	37 (100%)	74 (100%)	0.2785
Liver metastasis				
Yes	10 (27%)	11 (30%)	21 (28%)	0.7965
No	27 (73%)	26 (70%)	53 (72%)	
Surgery for primary lesion				
Yes and No	37 (100%)	37 (100%)	74 (100%)	0.3039
Radiation				
Yes	17 (46%)	16 (43%)	33 (45%)	0.8151
No	20 (54%)	21 (57%)	41 (55%)	
Chemotherapy				
Yes and No	37 (100%)	37 (100%)	74 (100%)	0.6433

Note: Due to NCDB agreement, cells with less than 10 cases in Race, Charlson-Deyo score, Brain metastasis and Chemotherapy were combined with other opposing cells

**Table 3:** Univariate and Multivariate Analysis in Original Cohort

Variable	Univariate HR (95%CI)	P value	Multivariate HR (95%CI)	P value
Age: <70/70+	0.75 (0.63-0.89)	0.0013	0.87 (0.73-1.05)	0.151
Sex: F/M	0.79 (0.67-0.93)	0.005	0.79 (0.67-0.94)	0.0063
Race: Other/W	0.78 (0.62-0.98)	0.0297	0.83 (0.65-1.05)	0.1199
Insurance: Other/Uninsured	0.88 (0.57-1.46)	0.6016	0.74 (0.47-1.23)	0.2318
Institution: Academic/Other	0.83 (0.69-0.98)	0.033	0.92 (0.76-1.11)	0.3896
Charlson-Deyo score: 0-1/2-3	0.79 (0.63-1.00)	0.047	0.84 (0.67-1.07)	0.1619
Brain metastasis: N/Y	0.95 (0.79-1.13)	0.5406	0.94 (0.77-1.16)	0.5829
Liver metastasis: N/Y	0.75 (0.63-0.90)	0.0026	0.81 (0.67-0.99)	0.0392
Surgery: Y/N	0.50 (0.32-0.73)	0.0001	0.46 (0.30-0.68)	<0.0001
Radiation: Y/N	0.91 (0.77-1.07)	0.248	0.98 (0.81-1.20)	0.8736
Chemotherapy: Y/N	0.44 (0.37-0.52)	<0.0001	0.44 (0.37-0.53)	<0.0001
Immunotherapy: Y/N	0.63 (0.42-0.91)	0.0112	0.64 (0.43-0.93)	0.0164

## Discussion

LCNEC is a relatively rare and aggressive type of lung cancer with abysmal prognosis that accounts for 3% of lung cancer with most patients being diagnosed in advanced stages (Fasano et al., 2015). LCNEC is classified accordingly because of its biological and clinical features. It is included in the group of thoracic neuroendocrine tumor per 2015 WHO classification of lung and pleural tumors (Travis et al., 2015).

Pulmonary LCNEC may have the following features which include (1) morphology of nesting, peripheral palisading, and rosettes (2) expression of neuroendocrine markers like synaptophysin, chromogranin A, TTF-1 Thyroid transcription factor, and CD56 (3) necrosis over large zones with mitotic rates >10 per 10 high power fields (Travis et al., 2015). Despite efforts to define these features, diagnosis of LCNEC remains a challenge for pathologists, especially with small biopsy specimens. For instance, in the two prospective, single-arm clinical trials of SCLC-like chemotherapy regimens for advanced LCNEC, central pathology review determined 11 of 41 cases and 11 of 40 cases should be reclassified as different diagnoses (Le Treut et al., 2013, Niho et al., 2013), demonstrating frequent disagreement among pathologists.

To improve diagnosis and understanding of LCNEC, researchers investigated molecular characteristics of LCNEC to further define its unique biologic features. Rekhtman et al., (2016) identified that 40% of these tumors are similar to SCLC which is characterized by p53 and RB1 gene alterations, whereas 55.5% had mutations such as STK11/Kras that are commonly seen in NSCLC. Moreover, 15% of LCNEC tumors showed genetic changes in P13K/AKT/mTOR pathway; it was also observed that LCNEC might have activating mutations in receptor tyrosine kinase genes such as EGFR, KIT, ERBB2 (Umemura et al., 2014, Miyoshi et al., 2017). Although these finding do not seem very helpful in current practice, further research into the molecular mechanisms of LCNEC might assist oncologists in the future.

More practically, medical oncologists facing advanced LCNEC in clinic must determine how to manage the cases with systemic therapy. Most reports below suggest using SCLC-based regimens in LCNEC patients. Sun et al. (2012) revealed that advanced LCNEC could be treated in a similar manner as SCLC rather than NSCLC and the response rates to platinum-based chemotherapy were 60% compared to non-platinum based chemotherapy which was 11%, with the overall survival OS being 16.5 vs. 9.2 months in SCLC regimen and NSCLC regimen group, respectively. 

Consistent with these findings, another study conducted by Shimada et al., (2012). observed a response rate of 61% vs. 63% to initial chemotherapy and that of 86% vs. 98% to chemoradiotherapy in patients high grade LCNEC vs. SCLC, suggesting that chemotherapy treatment using SCLC standard protocol significantly improves the OS of patients with LCNEC compared to that of NSCLC-based protocols.

In contrast, there are contradictory reports that do not suggest use of SCLC-like regimens. Igawa el al., (2010) evaluated 14 patients with high-grade unresectable LCNEC, with various platinum-based combination regimens or irinotecan (SCLC-like regimen) vs. vinorelbine or docetaxel alone (NSCLC-like regimen), and found the objective response rate to be 50% (7/14) vs. 53% (41/77); one-year survival rate to be 34% vs. 48%; median survival time of 10 months vs. 12.3 months. 

In keeping with this, another study conducted by Varlotto et al., (2011) based on the data obtained from Surveillance, Epidemiology and End Results Program (SEER) of the US National Cancer Institute, stated that in patients with LCNEC had characteristics where overall survival and Lung cancer-specific survival rates were more similar to those with other large cell carcinomas than SCLC. Derks et al., (2017) also reported overall survival for LCNEC patients treated with NSCLC based regimen was significantly longer than that for those treated with SCLC based regimen with a median survival of 8.5 vs 6.7 months, respectively. While this controversy still remains, prognosis of advanced LCNEC remains extremely poor regardless of treatment regimens. There is a need for novel systemic therapies to improve poor outcomes for patients with LCNEC. 

The use of IO agents has shown promising results in the treatment of solid tumors such as melanoma, NSCLC, renal cell cancer; therefore, we investigated IO use in LCNEC of the lung. Since first-line chemotherapy has limited efficacy in LCNEC, the use of IO may become an alternative option in the treatment of advanced LCNEC. PD-1/PD-L1 inhibitors are proven to improve survival in advanced stage NSCLC, and also have activity for SCLC (Horn et al., 2018, Paz-Ares et al., 2019). Still, IO efficacy in LCNEC is unknown and limited due to a few case reports. 

In the first case report, metastatic LCNEC in two patients confirmed by lung biopsies were treated with nivolumab as the sixth and third-line of treatment, showing responses in both cases with a decrease in serum tumor marker levels and significant tumor reduction (Daido et al., 2017). In a second case report, a strong and robust response to pembrolizumab was observed in a metastatic LCNEC despite the tumor being PD-L1 negative by immunohistochemistry (Wang et al., 2017). A third paper reported a case of locally advanced LCNEC with complete tumor response after palliative thoracic radiotherapy and treatment with nivolumab, indicating that radiation may enhance the activity of PD-1/PD/L1 inhibitors in LCNEC (Mauclet et al., 2019).

This retrospective NCDB analysis demonstrated that the IO group had 12 and 18-month survival of 34.0% and 29.1% as compared to 24.1% and 15.0% in the non-IO group. Multivariate analyses showed that female sex, absence of liver metastasis, use of surgery, use of chemotherapy, use of IO remained statistically significant. Propensity score matching analysis in overall survival showed a non-significant trend (p=0.0733) in favor of the IO group. These findings suggest that IO treatment benefits patients with advanced LCNEC.

We however acknowledge the limitation of current study. This is a retrospective, “hospital-based” data analysis using NCDB database. With lack of central review, histologic diagnosis of LCNEC was completely up to local pathologists. As discussed earlier, there might be cases to which alternative diagnoses can be assigned due to common discrepancies among pathologists. Administration of IO agents was recorded only when they were used as part of first course of therapy. Information regarding regimen, dose, frequency, duration, presence of other concurrent treatment modality was not available. It was unknown how IO agents were obtained for treatment such as through prospective clinical trials. The number of cases treated with IOs was relatively small, accounting for only 5.6% of total population. Nevertheless, the current study includes propensity score matching and a larger sample size than what is currently available in the literature. 

In conclusion, our findings suggest that use of IO might improve outcomes for advanced LCNEC patients. Further investigation is warranted to define the role of IO treatment in advanced LCNEC.

## Data Availability

The data supporting their findings were provided by National Cancer Database (NCDB). The data will not be shared due to NCDB’s policy. Financial Support and Sponsorship: not applicable Ethical approval and consent to participate Not applicable. This is a retrospective analysis on anonymized data provided by NCDB. The study was reviewed and approved by institutional review board at Parkview Health.

## References

[1] Daido W, Yamasaki M, Saito N, (in Japanese) (2017). Effectiveness of Nivolumab in large cell neuroendocrine carcinoma of lung – a report of two cases. Gan To Kagaku Ryoho.

[2] Derks JL, Jan van Suylen R, Thunnissen E (2017). Chemotherapy for pulmonary large cell neuroendocrine carcinomas: does the regimen matter?. Eur Respir J.

[3] Fasano M, Della Corte CM, Papaccio F, Ciardiello F, Morgillo F (2015). Pulmonary large-cell neuroendocrine carcinoma: from epidemiology to therapy. J Thorac Oncol.

[4] Horn L, Mansfield AS, Szczęsna A (2018). First-line atezolizumab plus chemotherapy in extensive-stage small-cell lung cancer. N Engl J Med.

[5] Igawa S, Watanabe R, Ito I (2010). Comparison of chemotherapy for unresectable pulmonary high-grade non-small cell neuroendocrine carcinoma and small-cell lung cancer. Lung Cancer.

[6] Le Treut J, Sault MC, Lena H (2013). Multicentre phase II study of cisplatin-etoposide chemotherapy for advanced large-cell neuroendocrine lung carcinoma: the GFPC 0302 study. Ann Oncol.

[7] Mauclet C, Duplaquet F, Pirard L (2019). Complete tumor response of a locally advanced lung large-cell neuroendocrine carcinoma after palliative thoracic radiotherapy and immunotherapy with nivolumab. Lung Cancer.

[8] Miyoshi T, Umemura S, Matsumura Y (2017). Genomic Profiling of Large-Cell Neuroendocrine Carcinoma of the Lung. Clin Cancer Res.

[9] Niho S, Kenmotsu H, Sekine I (2013). Combination chemotherapy with irinotecan and cisplatin for large-cell neuroendocrine carcinoma of the lung: a multicenter phase II study. J Thorac Oncol.

[10] Paz-Ares L, Dvorkin M, Chen Y (2019). Durvalumab plus platinum–etoposide versus platinum–etoposide in first-line treatment of extensive-stage small-cell lung cancer (CASPIAN): a randomised, controlled, open-label, phase 3 trial. Lancet.

[11] Rekhtman N (2010). Neuroendocrine tumors of the lung: an update. Arch Pathol Lab Med.

[12] Rekhtman N, Pietanza MC, Hellmann M (2016). Next-generation sequencing of pulmonary large cell neuroendocrine carcinoma reveals small cell carcinoma-like and non-small cell carcinoma-like subsets. Clin Cancer Res.

[13] Rosenbaum PR 1989) Optimal matching for observational studies. J Am Stat Assoc.

[14] Shimada Y, Niho S, Ishii G (2012). Clinical features of unresectable high-grade lung neuroendocrine carcinoma diagnosed using biopsy specimens. Lung Cancer.

[15] Sun JM, Ahn MJ, Ahn JS (2012). Chemotherapy for pulmonary large cell neuroendocrine carcinoma: Similar to that for small cell lung cancer or non-small cell lung cancer?. Lung Cancer.

[16] Travis WD, Bambilla E, Nicholson AG (2015). The 2015 World Health Organization Classification of Lung Tumors: Impact of Genetic, Clinical and Radiologic Advances Since the 2004 Classification. J Thorac Oncol.

[17] Travis WD, Linnoila RI, Tsokos MG (1991). Neuroendocrine tumors of the lung with proposed criteria for large-cell neuroendocrine carcinoma An ultrastructural, immunohistochemical, and flow cytometric study of 35 cases. Am J Surg Pathol.

[18] Umemura S, Mimaki S, Makinoshima H (2014). Therapeutic priority of the PI3K/AKT/mTOR pathway in small cell lung cancers as revealed by a comprehensive genomic analysis. J Thorac Oncol.

[19] Varlotto JM, Medford-Davis LN, Recht A (2011). Should large cell neuroendocrine lung carcinoma be classified and treated as a small cell lung cancer or with other large cell carcinomas?. J Thorac Oncol.

[20] Wang VE, Urisman A, Albacker L (2017). Checkpoint inhibitor is active against large cell neuroendocrine carcinoma with high tumor mutation burden. J Immunother Cancer.

[21] Zhang JT, Li Y, Yan LX (2020). Disparity in clinical outcomes between pure and combined pulmonary large cell neuroendocrine carcinoma: A multi-center retrospective study. Lung Cancer.

